# Supplementary material for: Research on the coordination of fiscal structure and high-quality economic development: An empirical analysis with the example of Anhui province in China
Source: PLoS One. 2023 Jun 15;18(6):e0287201. doi: 10.1371/journal.pone.0287201 (PMC10270635; doi:10.1371/journal.pone.0287201)
Supplement: Attachment — Submitted filename: Response to Reviewers.pdf [file pone.0287201.s002.pdf]

Dear editor and reviewers,
We are very grateful for your valuable comments. Our modifications are described as follows:

Editor:

1. Please review your reference list to ensure that it is complete and correct. If you have cited papers that have been retracted, please include the rationale for doing so in the manuscript text, or remove these references and replace them with relevant current references. Any changes to the reference list should be mentioned in the rebuttal letter that accompanies your revised manuscript. If you need to cite a retracted article, indicate the article's retracted status in the References list and also include a citation and full reference for the retraction notice.

***Response*:**
Our references are in compliance with the criteria, where reference [31] is in Table 3. Because Table 3 is too large, it is not in the manuscript but is uploaded as an attachment.

2. - please Correct spelling and grammar mistakes
   - please Add recent references
   - The literature review is modest, you will need to analyse more recent research related to the second,Fiscal structure and coordinated economic development , the third,Fiscal structure and green economic development, the fourth,Fiscal structure and open economic development and the fith, areas (pages 10,11,12).

***Response*:**

Thank you very much Dr. Bechir for raising these detailed questions. We are very sorry for not formally responding to your comments in the first revision, which were duplicated by your comments and those of the reviewers, and we have made changes. All changes in our paper are marked in red in the Revised Manuscript with Track Changes.docx file.
We have made changes to grammar and other issues.
We conducted a review of the relevant literature in the introduction, citing 27 recent papers. The main body of our paper is an analysis of the coherence between fiscal structure and quality economic

development. We have constructed the indicators of high-quality economic development based on the characteristics of China's economy in five aspects: innovation, coordination, green, openness and sharing. Most of the papers focus on the general analysis, and we are further thinking in five areas. As a result, there is relatively little literature in this area.

3. - the authors should clarify the strategic and operational implications

The fifth part of our manuscript is the conclusion and policy recommendations, which are based on the empirical findings. And we have added a reflection on the limitations of the paper.

Reviewer #2: the autors have made the requested corrections: he has corrected the litterarure review and added the implications

Reviewer #3: * The authors study this problematic: Research on the Coordination of Fiscal Structure and High-Quality Economic Development: An Empirical Analysis with the Example of Anhui Province in China.

* I consider that the problem is original and recent.

***Response*:**

Thank you again for your previous comments. And thank you for your approval of the revised manuscript.
