# Supplementary material for: Research on the coordination of fiscal structure and high-quality economic development: An empirical analysis with the example of Anhui province in China
Source: PLoS One. 2023 Jun 15;18(6):e0287201. doi: 10.1371/journal.pone.0287201 (PMC10270635; doi:10.1371/journal.pone.0287201)
Supplement: Attachment — Submitted filename: Response to Reviewers.docx [file pone.0287201.s001.docx]

Dear professor,

We are grateful to the reviewers for finding the paper of some value and providing valuable comments. We have revised the paper in accordance with the reviewers' comments.

Reviewer #1: The article is very interesting but some improvements are needed:
-Add recent references
-Correct spelling and grammar mistakes
-Add a literature review to the article
-Add a conclusion to the article

***Response*:**

Thank you very much for the suggestions you gave us, we have revised the paper as follows, adding recent references as well as a literature review. We have rechecked the grammar and spelling and corrected any errors. Also, the fifth part of our paper is the conclusion and policy recommendations.

All changes in our paper are marked in red in the Revised Manuscript with Track Changes.docx file.

Reviewer #2: -This research paper discusses an interesting problem of "measuring the index system of the fiscal structure and high-quality economic development in Anhui using the entropy weight method and and empirically analyzes the coordinated development
level of fiscal structure and high-quality economic development in Anhui using coupled coordination degree model". The literature review is modest, the authors will need to analyse more recent research related to the second,Fiscal structure and coordinated economic development , the third,Fiscal structure and green economic development, the fourth,Fiscal structure and open economic development and the fith, areas (pages 10,11,12)

***Response*:**

Thank you very much for your approval of our paper and for your valuable comments, we have added the latest literature on the five areas you provided. And the additions are marked in red.
